# Assessment of frequency response techniques in diagnosing polymer electrolyte membrane fuel cells

**DOI:** 10.1016/j.isci.2024.110254

**Published:** 2024-06-13

**Authors:** Antonio Sorrentino, Kai Sundmacher, Tanja Vidakovic-Koch

**Affiliations:** 1 Max Planck Institute for Dynamics of Complex Technical Systems, Magdeburg, Germany; 2 Otto-von-Guericke University Magdeburg, Process Systems Engineering, Magdeburg, Germany

**Keywords:** Electrochemistry, Engineering, Energy systems

## Abstract

In this study, we evaluated the effectiveness of various frequency response analysis (FRA) techniques for identifying fault states in the diagnosis of polymer electrolyte membrane fuel cells (PEMFCs). To this end, an identifiability analysis was conducted to determine the reliability of parameters obtained by fitting a previously validated PEMFC model to the spectra from different methods. Specifically, we focused on electrochemical impedance spectroscopy (EIS) and the newly introduced concentration frequency response analysis (CFRA). The identifiability analysis revealed that CFRA, when applied with water pressure as the input and voltage as the output, provides the most accurate parameters estimates related to mass transport in the cathode electrode and the Nafion electrolyte, yielding physically meaningful insights. Consequently, employing this input for PEMFC diagnosis emerges as a promising approach. Furthermore, our findings underscore the importance of meticulously evaluating the quality of parameter estimation, even when utilizing well-established techniques such as EIS.

## Highlights

• Performance of different frequency response methods for PEMFC diagnosis is evaluated
• The quality of estimated parameters is evaluated through identifiability analysis
• Use of water pressure inputs implies the most reliable parameter estimation

## Graphical abstract

**Figure:**
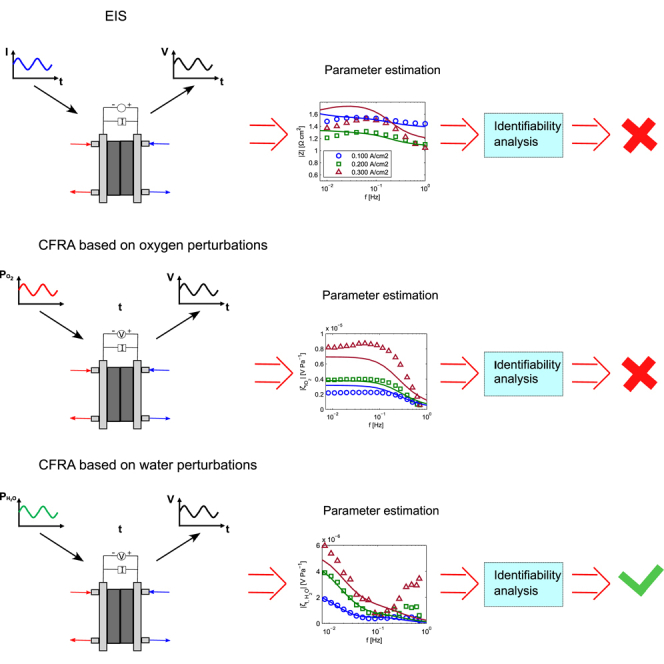

## Introduction

The establishment of a hydrogen economy is at the center of the energy policy of the most industrialized countries that aim to reduce the ${\text{CO}}_{2}$ emissions.^1^^,^^2^ In this scenario, polymer electrolyte membrane fuel cell (PEMFC) is seen as a key technology to empower electric vehicles, especially heavy duty ones.

In order to be suitable for commercialization, the European Commission set the requirement of 7,000 h and 28,000 h as the lifetime for PEMFC stacks employed in cars and buses, respectively. However, the corresponding international state-of-art lifespans are only 4,000 h and 16,000 h.^2^

A way to increase the durability of a PEMFC system is to complement it with a mitigation strategy that prevents the rise of faulty states accelerating degradation mechanisms. This implies a real-time monitoring of the stack through an experimental routine enabling the identification of specific faulty states. The most employed experimental technique for such use is the electrochemical impedance spectroscopy (EIS), as it allows to differentiate the contributions of different physicochemical phenomena and degradation processes from the others.^3^^,^^4^^,^^5^^,^^6^^,^^7^

However, the analysis of EIS data is often not easy due to the coupling of the effects of different processes in the spectra. Different strategies have been proposed to overcome this problem. Some groups have proposed to extend the EIS analysis from linear to nonlinear range. Basically, an electric periodic input characterized by an amplitude higher than the one usually used in linear EIS is applied to the cell. The complementary electric response contains non-linearities that are analyzed by harmonic analysis means. In this way, different phenomena occurring in PEMFCs could be discriminated from each other.^8^ A general review on this method is given by Vidakovic-Koch et al.,^9^ while one more specific on the application on PEMFC is reported by Yuan et al..^10^

Another strategy to overcome the issue of the EIS is the development of alternative frequency response techniques based on the use of non-electrical inputs and/or outputs. These techniques aim to analyze the contribution to the power losses of a specific phenomenon or provide additional information about state of health of the PEMFC. For example, techniques based on the modulation of back pressure,^11^^,^^12^^,^^13^ partial pressure of the feeds,^14^^,^^15^^,^^16^^,^^17^ and temperature^18^ have been developed. One of the advantages of some of these methodologies is also the cheaper equipment compared to the one required for application of a classic EIS. A concise review about these new techniques has been published by the authors of this paper.^19^ More recently, a general prospective regarding their use for different types of electrochemical devices has also been given by Kadyk et al.^20^

Among the others, frequency response analysis (FRA) methods based on modulated inputs of oxygen and water partial pressures sent to the cathode, also named concentration-alternating frequency response analysis (CFRA), attracted the interest of several research groups due to the possibility to decouple the dynamics of gas transport and water transport in the Nafion membrane from the others.^14^^,^^17^ Since they could potentially improve the robustness of the diagnosis of PEMFC systems, several theoretical studies deepening the implications of this novel approach have been published in the last years.^21^^,^^22^^,^^23^^,^^24^^,^^25^

A mitigation strategy also requires a model in order to analyze the data and diagnose the type of fault detected. Generally, white box and gray box physically based models are employed. While the former model type, constituted by a set of partial differential equations (PDEs) and nonlinear algebraic relations, is more detailed and computationally expensive; the latter ones are simplified versions containing empirical or semi-empirical algebraic relations to describe some variables or the dependence of some parameters on the operating conditions. For this reason, the gray box models are characterized by lower computational times and, therefore, they are more preferable candidates for the use in diagnostic tools.^3^^,^^5^^,^^6^

**Figure 1:**
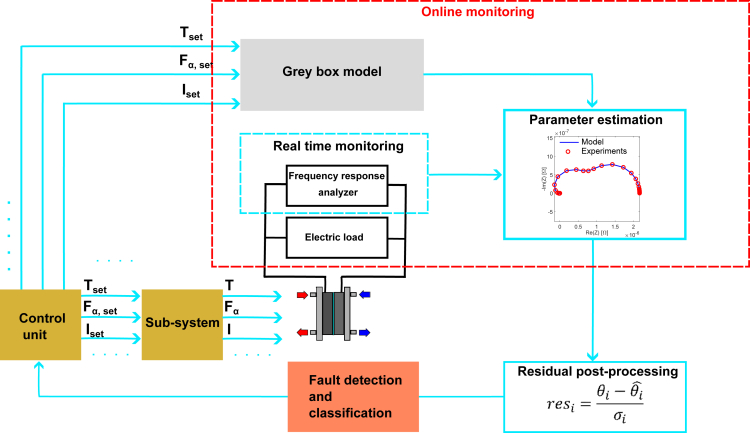
Schematic depicting a fault detection and isolation architecture for PEMFCs diagnosis based on EIS The online monitoring is highlighted, as it is the section of interest in this study.

Figure 1 depicts how an FRA technique and a gray model are integrated in the online monitoring unit of an identification and isolation architecture of a PEMFC. As displayed, FRA spectra are collected at different time intervals from the PEMFC during operating conditions and fitted to the gray box model. The obtained parameters are confronted to nominal ones previously determined by fitting spectra of the cell in healthy operating conditions. In case the residual of one or some of them exceeds a fixed threshold, a classification algorithm processes the data and identifies the faulty states. The information is sent to a control unit that changes the inputs of the subsystem accordingly (electric load, fuel conditioning etc.) to mitigate the negative consequence. The success of this diagnosis scheme is, then, dependent on the trustworthiness and quality of the parameter estimated in the online monitoring unit.

Considering this framework, in this work, the performance of EIS and CFRA employed as tools in the online monitoring are benchmarked. This is done by evaluating the quality of the parameter estimated by fitting of their respective spectra through an identifiability analysis. In this way, we could verify if the use of frequency response methods based on concentration perturbations implies more robust diagnosis routines than the ones based on EIS.

The work is organized as follows. In the section methods, a summary on the capabilities of the considered CFRA techniques in comparison to one of EIS is given. Then, the PEMFC model employed in the analysis and the identifiability procedure are described. In the section results and discussion, the identifiability of parameters estimated by fitting of spectra of each considered frequency response methods is evaluated. Finally, the statements of the identifiability analysis are validated by verifying the plausibility of parameters obtained by fitting of the FRA spectra at different operating conditions. A comparison between the estimated parameters and the ones measured in the literature is also included.

### Methods

#### Brief description of CFRA techniques and comparison to EIS

**Figure 2:**
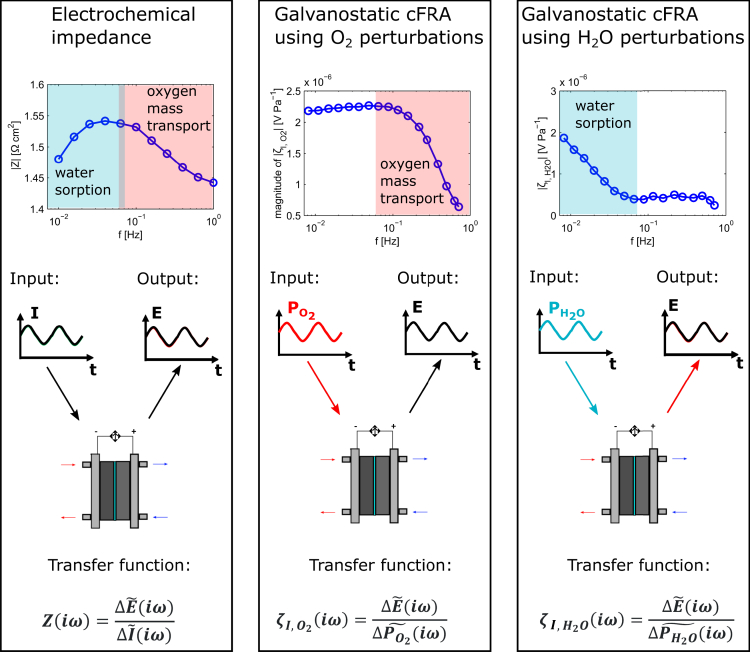
Schematic representation summarizing the differences between EIS and CFRA spectra

A schematic describing the FRA considered in this work and showing the corresponding spectrum features is depicted in Figure 2. Unlike the EIS, which involves an electrical input (generally current) and output (generally voltage), the perturbation in the CFRA consists of a flow rate with a periodic variation of the partial pressure of reactants over time. The electrical response depends on the type of control applied to the PEMFC, i.e., galvanostatic or voltastatic.^14^^,^^17^

In this article, we consider oxygen and water partial pressure perturbations sent to the cathode side under galvanostatic control. Therefore, the related transfer functions are constituted by the ratio between the Fourier transform of the voltage outputs and partial pressures inputs at different frequencies. In some studies, the outlet partial pressures as response to applied sinusoidal voltages are collected.^25^ This technique is named concentration admittance. In that case, the obtained transfer function is the reciprocal of one of the CFRA and contains the same information. Therefore, all the findings reported in this study should be theoretically valid for both methodologies.

As reported in our previous studies,^14^ the frequency range of the processes that can be analyzed by CFRA is limited due to the fact that the partial pressure perturbation has to propagate from the channel and gas diffusion layer (GDL) to the catalyst layer before having an effect on the electric response. It means that only the effects of transients with a comparable or lower time constants of the gas transport in the channel and GDL can be observed in the spectra. Additionally, technical features of the experimental setups used so far to perform the CFRA experiments limit the highest frequency measurable at 1 Hz.^17^

In the spectra of Figure 2, the main findings from our previous study,^17^ which combines modeling and experiments, are summarized. Among different representations, the Bode magnitude plot is chosen to visualize the data due to less sensitivity to the measurement noise. As can be seen in Figure 2, all the dynamics acting in the frequency range 1–0.01 Hz, i.e., mass transport in the channel and water sorption in the membrane, are detected by performing EIS. In the CFRA spectra obtained using oxygen perturbations under galvanostatic control, only the dynamics of gas transport in the channel is observed. On the other hand, only the contribution of the water sorption in Nafion is visible using water partial pressure as periodic input. Therefore, CFRA can be utilized to selectively monitor the impact on the PEMFC performance of these processes. Moreover, it can facilitate the interpretation of the EIS spectra in cases where a strong coupling between different processes is verified. Details about the capabilities of the CFRA and the ones of similar methods like concentration admittance can be found in other studies.^14^^,^^17^^,^^21^^,^^22^^,^^23^^,^^24^^,^^25^

#### PEMFC model

A PEMFC model developed and validated in our previous studies is employed in this work.^17^ It is a simplified one-dimensional and isothermal formulation. The main equations are displayed in Table 1. Dynamic mass balances are formulated for all chemical species present in each of the compartments constituting the PEMFC. Different assumptions have been made depending on the features of the domains and the type of transport mechanism. For instance, the channel is treated as perfectly mixed (ideally stirred reactor). Fickian-type diffusion is assumed to describe the gas transport inside the GDL. The mass balance for the water in Nafion is based on the assumption that the water is in liquid state in the membrane. Moreover, Fickian-type diffusion and electro-osmotic drag are contemplated as transport mechanisms.

**Table 1:** Main equations of the PEMFC model

**Mass balance in the channels**

$V\frac{dP_{\alpha }^{\text{CH}}}{\text{dt}}=P_{\alpha ,\text{in}}^{\text{CH}}F_{\text{in}}-P_{\alpha }^{\text{CH}}F_{\text{out}}-\text{RT}A_{\text{cell}}J_{\alpha }{|}_{\text{CH}/\text{GDL}}$
$F_{\text{out}}=F_{\text{in}}-\frac{\text{RT}A_{\text{cell}}}{P_{\text{tot}}}\cdot \sum_{\alpha =1}^{n}J_{\alpha }{|}_{\text{CH}/\text{GDL}}$
$J_{\alpha }{|}_{\text{CH}/\text{GDL}}={-\frac{D_{\alpha }^{\text{eff}}}{\text{RT}}\frac{\partial P_{\alpha }^{\text{GDL}}}{\partial x}|}_{\text{CH}/\text{GDL},t}$

**Mass balance in the GDL**

$\frac{\partial P_{\alpha }^{\text{GDL}}}{\partial t}=D_{\alpha ,\beta }^{\text{eff}}\frac{\partial^{2}P_{\alpha }^{\text{GDL}}}{\partial x^{2}}$
$D_{\alpha ,\beta }^{\text{eff}}=\varepsilon_{\alpha }^{1.5}D_{\alpha ,\beta }$

**Mass balance in Nafion membrane**

$\frac{\rho_{\text{EW}}}{m_{\text{EW}}}\frac{\partial \lambda_{H_{2}O}^{M}}{\partial t}=\frac{\partial \left(D_{H_{2}O}^{M}\left(\lambda_{H_{2}O}^{M}\right)\frac{\rho_{\text{EW}}}{m_{\text{EW}}}\frac{\partial \lambda_{H_{2}O}^{M}}{\partial x}+\xi \left(\lambda_{H_{2}O}^{M}\right)\frac{j}{F}\right)}{\partial x}$
$D_{H_{2}O}^{M}\left(\lambda_{H_{2}O}^{M}\right)=D_{H_{2}O}^{0,M}\lambda_{H_{2}O}^{M}\;\exp\left(-\frac{2436}{T}\right)$
$\xi \left(\lambda_{H_{2}O}^{M}\right)=K_{H^{+}}\lambda_{H_{2}O}^{M}$

**Charge balance at cathode catalyst interface**

$C_{c}^{\text{DL}}\frac{d\eta_{c}}{\text{dt}}=-j+4Fr_{\text{ORR}}$
$r_{\text{ORR}}=\frac{P_{O_{2}}^{\text{CL}}}{\text{RT}C_{O_{2},\text{ref}}}t_{c}^{\text{CL}}ai_{0}\;\exp\left(-\frac{\alpha_{c}F}{\text{RT}}\eta_{c}\right)$

Charge balances are formulated at the catalyst layer, which is considered as a thin interface between the GDL and the Nafion membrane (for the sake of simplicity, the anode charge balance has been left out). The kinetics of hydrogen oxidation (data not shown) and oxygen reduction are lumped; side reactions such as platinum oxide and hydrogen peroxide formation were not taken into account. A dynamic mass balance for the liquid water in the GDL is not contemplated. The simplified mass balance for the channel limits the validity of the model under high stoichiometry values of the feeds. Further details about the model assumptions can be found in a study by Sorrentino et al.^14^ The meaning of all the symbols used can be found in the table displayed in the STAR Methods section.

Despite the many simplifications, the model simulations reproduce with good agreement experimental EIS and CFRA spectra that were collected using feed characterized by low relative humidity and relatively high stoichiometry values. As pointed out in many other studies,^11^^,^^26^^,^^27^ three processes mostly influences the transient behavior of a PEMFC under these operating conditions: (1) charging and discharging of the double layer, (2) mass transport of the gaseous reactants in the channel/GDL, and (3) water sorption into the Nafion. The model supports this interpretation.

We regard this model as a good trade-off between the level of detail (complexity) and description of experimental observations. Moreover, its relatively low computational time (less than 2 s for computation of one FRA spectrum on an ordinary laptop) makes it suitable for employment as a gray box model in online fault diagnosis and identification architectures. Consequently, it constitutes a good example for the purpose of this article.

### Identifiability analysis procedure

Let us consider a model that correctly describes the physical behavior of a system. The goal of identifiability analysis is to find out if a unique set of model parameters can accurately fit a specific dataset. In other words, it assesses the reliability of estimated parameters for evaluating the properties of the real system. If the identifiability is poor, it means there are multiple parameter sets that can fit the data equally well, making it impossible to pinpoint the “true” parameter values. Alternatively, conducting an identifiability analysis for a model can help determine if the dataset is appropriate for parameter estimation. In this regard, the optimal dataset would imply an orthogonal influence of the parameters on the objective function of the fitting algorithm. Moreover, the parameters of a model can also be partially identifiable, meaning that a unique solution can only be determined for a subset of them. For a more comprehensive introduction to this topic, one can refer to the textbook written by Belsey.^28^

Among other methodologies, this study applies a framework commonly known as practical identifiability.^28^^,^^29^^,^^30^^,^^31^^,^^32^^,^^33^ This analysis has been widely employed in the field of chemical process engineering and system biology to analyze the kinetics of complex chemical networks.^30^^,^^31^^,^^32^ Recently, it has been applied to assess the identifiability of parameters obtained by fitting of a PEMFC model to polarization curves under different experimental conditions.^34^ To our knowledge, this is the first case where the following analysis is used to benchmark the reliability of parameters estimated by fitting data of different dynamic techniques.

Let us consider a general transfer function H and the related vectors $H_{\exp}={\left(H_{\exp,1},\ldots H_{\exp,n}\right)}^{T}$ and $H_{\text{sim}}\left(\theta \right)={\left(H_{\text{sim},1}\left(\theta \right),\ldots H_{\text{sim},n}\left(\theta \right)\right)}^{T}$ containing, respectively, its experimentally measured values $H_{\exp,i}$ at different frequencies $\omega_{i}$ and the predicted ones $H_{\text{sim},i}$ from a considered model using the parameter set $\theta ={\left(\theta_{1},\ldots \theta_{m}\right)}^{T}$. The relationship between them reads:

(Equation 1) $$H_{\exp}=H_{\text{sim}}\left(\theta \right)+L\text{.}$$

where $L={\left(L_{1},\ldots L_{n}\right)}^{T}$ is the error vector whose elements quantify the deviation between the experiments and model predictions. While relevant for assessing the adequacy of the estimated parameters, this is not crucial for their identifiability; thus, its value is of lesser importance for our analysis.

Then, let us consider a subset K among all the model parameters set θ. To be identifiable, it must fulfill two requirements. First, the simulated outputs $H_{\text{sim}}\left(\theta \right)$ must be sensitive to the change of all parameters of the subset. Second, the change of the output due to the modification of a parameter should not be compensated by the change of value of another considered parameter. The first criterion is addressed by the determination of the sensitivity coefficients of each parameter of the chosen subset and their corresponding mean square value $\delta_{j}^{\text{msqr}}$. The second is evaluated by calculating the collinearity index.

To determine both of them, the elements $s_{i,j}$ of the non-dimensional sensitivity matrix $S_{p}$ have to be calculated according to the following equation:

(Equation 2) $$s_{i,j}=\frac{\theta_{j}^{0}}{H_{\text{sim},i}^{0}}\frac{\partial H_{\text{sim},i}}{\partial \theta_{j}}\text{.}$$

where the index i stands for the value of the considered transfer function at a certain frequency $\omega_{i}$, while the index j indicates the parameter considered. As a local measure of sensitivity, the derivative in Equation 2 is determined with respect to a fixed initial parameter set $\theta_{0}$. Consequently, the elements $H_{\text{sim},i}^{0}$ and $\theta_{j}^{0}$ are respectively the output of the model computed employing the parameter set $\theta_{0}$.

From the elements of $S_{p}$, the mean square sensitivity $\delta_{j}^{\text{msqr}}$ related to a certain parameter $\theta_{j}$ is then determined through the following equation:

(Equation 3) $$\delta_{j}^{\text{msqr}}=\sqrt{\frac{1}{N}\sum_{i=1}^{N}s_{i,j}^{2}}\text{.}$$

where N is the number of the elements of the dataset. In our case, N is the number of frequency points $\omega_{i}$ at which the transfer function is measured. While the elements of the sensitivity matrix $s_{i,j}$ give a measure of the sensitivity for a parameter $\theta_{j}$ at certain frequency $\omega_{i}$, the value $\delta_{j}^{\text{msqr}}$ is an overall determination of the sensitivity of the transfer function to the j-th parameter at all the collected frequency points.

A high value of the mean square sensitivity for a certain parameter implies a low statistical variance and, consequently, its estimation is less influenced from the noise eventually present in the measurements. On the other hand, a lower mean square sensitivity means a larger variance for the fitted parameter and, therefore, a bigger uncertainty regarding its value.

To calculate the collinearity index of a parameter subset K, the non-dimensional sensitivity matrix must be computed in its normalized form ${\tilde{S}}_{p}$. Then, the elements of this matrix read:

(Equation 4) $${\tilde{s}}_{i,j}=\frac{s_{i,j}}{\left\|s_{j}\right\|}\text{.}$$

Here, $\left\|s_{j}\right\|$ represents the Euclidean norm of the j-th column of the non-dimensional sensitivity matrix $S_{p}$. Finally, the collinearity index $\gamma_{K}$ of the parameter subset K is determined according to the following equation:

(Equation 5) $$\gamma_{K}=\frac{1}{\min\left(\text{eigen}\left({{\tilde{S}}_{p,K}}^{T}{\tilde{S}}_{p,K}\right)\right)}=\frac{1}{\lambda_{K}}$$

where ${\tilde{S}}_{p,K}$ is a sub-matrix of ${\tilde{S}}_{p}$ containing only the columns related to the parameters of the subset K, while $\lambda_{K}$ is the smallest eigenvalue of ${{\tilde{S}}_{p,K}}^{T}{\tilde{S}}_{p,K}$.

Basically, the collinearity index $\gamma_{K}$ is a measure of the degree of linear dependence of the columns in ${\tilde{S}}_{p,K}$. Its value can vary in a range from 1 to infinite. The former limiting case indicates the orthogonality of the columns of the subset K. If it is verified, a certain change in the values of the simulated transfer function is related to a unique variation of the parameters set. As a consequence, upon the running of an optimization algorithm for parameters fitting, the cost function converges to a unique solution. The parameters are then identifiable. On the contrary, for big values of the collinearity index, the columns of ${\tilde{S}}_{p,K}$ exhibits a linear dependence for some extent. In this case, the use of an indefinite number of parameters sets implies the same variation of the simulated transfer function. As a result, the optimization algorithm does not converge to a unique solution, rendering the parameters unidentifiable. This can also be visualized by observing saddle points in the contour plot of the cost function for parameter fitting.^35^^,^^36^

A value of 10 for $\gamma_{K}$ has been indicated by Belsley^28^^,^^29^ as the limit above which the collinearity between the parameters of the subset is not tolerable and the identification of them is not possible. This value is used in many studies.^30^^,^^31^^,^^32^ In this work, this threshold value is also assumed.

## Results and discussion

In this section, the identifiability of parameters related to typical faulty states of PEMFCs is evaluated combining sensitivity and collinearity analysis described in the previous section. The analysis considers experiments presented in a previous work by us.^17^ In that work, we collected EIS and CFRA spectra at three different steady-state current densities. Further information on the experimental conditions can be found in a study by Sorrentino et al.^17^

As this is a local identifiability analysis, a suitable initial parameter set $\theta_{0}$ must be defined. We considered for it the parameters estimated in our previous study,^17^ as they could reproduce the experimental FRA spectra with a good qualitative and quantitative agreement at all the experimental conditions. Moreover, their values are comparable to the ones present in the literature.^37^^,^^38^ More details can be found in the relative table displayed in the STAR Methods section.

It must also be remarked that the initial parameter set influences the results of the identifiability analysis, and in some cases could lead to different conclusions. A study of the effects of choosing different initial conditions is, however, out of the scope of this work. Here, the focus is on evaluating the trustworthiness of a fault identification scheme for PEMFCs. For this application, a single nominal parameter set has to be fixed among the others. An important requirement for this is the good agreement between the simulations and the experimental data at nominal conditions employing the selected parameters. This requirement is met and, therefore, the analysis is only performed considering such set of initial parameters.

Degraded catalyst, flooding of the electrode at the cathode side, and membrane drying out have been considered as faulty conditions. Their emergence has been respectively related to the values of following parameters: (1) the charge transfer coefficient $\alpha_{c}$, (2) the porosity relative to the effective diffusion of oxygen $\varepsilon_{O_{2}}$ and water $\varepsilon_{H_{2}O}$ inside the cathode GDL, and (3) the intrinsic diffusivity of the water in Nafion $D_{H_{2}O}^{0,M}$ and the electro-osmotic constant $K_{H^{+}}$ (see the mass balance in Nafion membrane displayed in Table 1). $\varepsilon_{O_{2}}$ and $\varepsilon_{H_{2}O}$ constitute artificial parameters. They were introduced to scale the values of the fitted diffusivities with respect to the nominal values $D_{O_{2}}^{\text{eff}}$ and $D_{H_{2}O}^{\text{eff}}$. The parameters $D_{H_{2}O}^{0,M*}$ and $K_{H^{+}}^{*}$ are also scaled with respect to their nominal values in the fitting procedure.

### Identifiability analysis

The elements of the non-dimensional sensitivities matrix $s_{i,j}$ have been calculated numerically according to the following equation:

(Equation 6) $$s_{i,j}=\frac{\theta_{j}^{0}}{\left|H_{\text{sim},i}^{0}\right|}\frac{\left|H_{\text{sim},i}\left(\theta_{j}^{0}+\Delta \theta_{j}\right)\right|-\left|H_{\text{sim},i}^{0}\right|}{\Delta \theta_{j}}\text{.}$$

where $\Delta \theta_{j}$ is a small arbitrary incremental quantity added to the parameter $\theta_{j}$. Among the other representation of the transfer function H, the sensitivities relative to the magnitude |H| have been determined. In order to verify the accuracy of the approximation of the first derivative in the Equation 6, the calculations have been repeated at any time using the multiple of the incremental value $2\Delta \theta_{j}$. If the value was found to be equal to the one previously computed, then the approximation was considered correct. Otherwise, the sensitivity was recalculated using half of the initial increment until this condition of equality was met.

**Figure 3:**
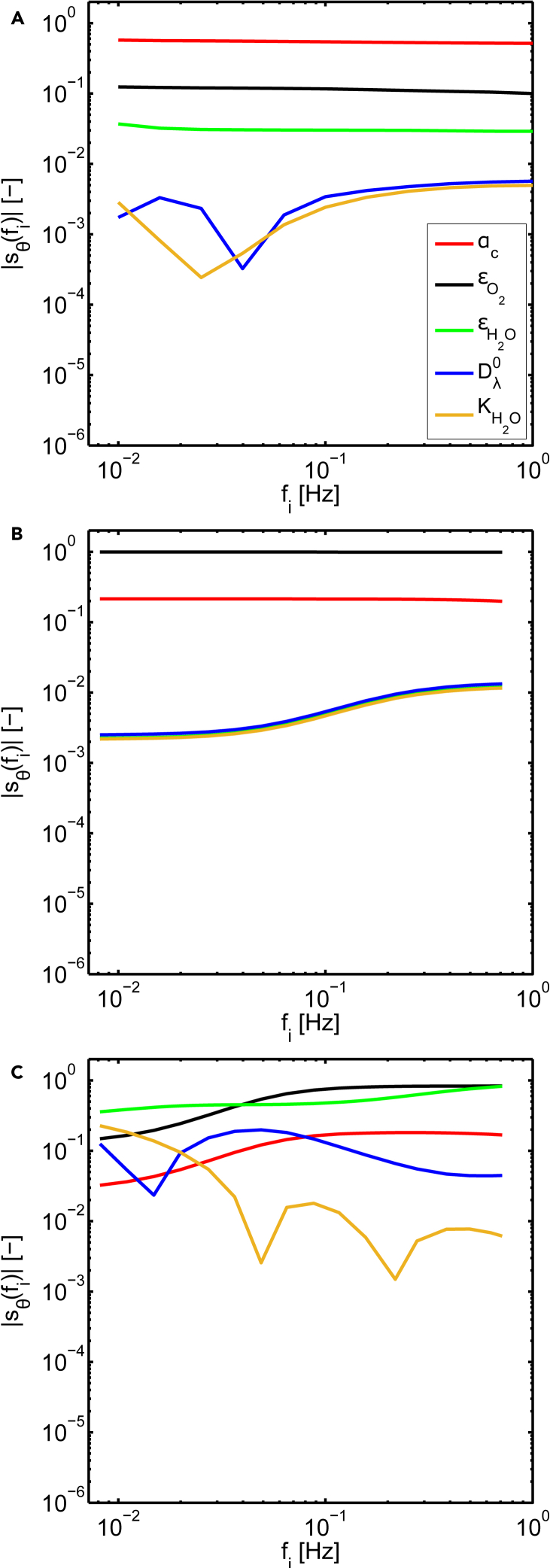
Non-dimensional sensitivities for different FRA methods (A) EIS. (B) CFRA performed through oxygen pressure perturbations. (C) CFRA performed through water pressure perturbations. Steady state current: 100 mA ${\text{cm}}^{-2}$

The non-dimensional sensitivities to the selected parameters of all the considered FRA transfer functions at 100 mA ${\text{cm}}^{2}$ are displayed in Figures 3A–3C. The sensitivities determined at other operating current densities differ only quantitatively from the ones shown and, for this reason, the following statements are valid also in these other cases.

The highest sensitivity is observed for the charge transfer coefficient and diffusivity of oxygen in GDL in the EIS and CFRA through oxygen pressure input. This trend holds along all the considered frequency range. On the other hand, these techniques present an extremely low sensitivity to all parameters related to the water transport. The sensitivity to $\varepsilon_{H_{2}O}$ overlaps with the ones of the parameters related to the transport in Nafion in the case of the CFRA through oxygen inputs, and it is, for this reason, not visible in Figure 3. On the contrary, CFRA obtained by water pressure inputs shows significantly higher sensitivity values for such parameters. This last finding confirms the suppositions advanced in previous works about the particular suitability of the use of dynamic water pressure inputs to detect phenomena related to membrane hydration.^14^^,^^15^^,^^17^

It is noticeable that the sensitivities to the charge transfer coefficient and oxygen effective diffusivity are proportional for all the FRA techniques. This could indicate a strong correlation among these parameters and the impossibility of a reliable estimation of them by fitting of a single spectrum. The same proportionality relationship is observed for the sensitivities to the water diffusivity in Nafion and electro-osmotic drag constant, but only for the EIS and CFRA through oxygen pressure perturbation. Additionally, this trend is verified only at higher frequencies in the EIS (f $>\text{rbin}10^{-1}$ Hz). On the other hand, the qualitative trends of the sensitivities of the CFRA using water pressure perturbation to the water transport parameters is unrelated all over the frequency region considered. This could mean a low interdependence among these parameters and, consequently, a successful identification of them. The implications of these findings are further deepened through the analysis of the collinearity index.

The mean square sensitivities expressing the overall influence of a parameter on the simulated magnitude plots are displayed in Table 2. Unlike the previous diagram, the values at all the steady state current densities are reported. The trends previously observed for the local sensitivities are verified for the corresponding mean squared values as well. However, the tendency along the different operating current densities provides additional information about the computed FRA experiments.

**Table 2:** Mean square sensitivities of the different transfer functions at three steady state currents

Current	Technique	$\delta_{\alpha_{C}}^{\text{msqr}}$	$\delta_{\varepsilon_{O_{2}}}^{\text{msqr}}$	$\delta_{\varepsilon_{H_{2}O}}^{\text{msqr}}$	$\delta_{D_{H_{2}O}^{M}}^{\text{msqr}}$	$\delta_{K_{H^{+}}}^{\text{msqr}}$
100 mA ${\text{cm}}^{-2}$	EIS	0.58	0.11	0.03	0.004	0.003
	CFRA($O_{2}$) gal.	0.99	0.19	0.007	0.008	0.007
	CFRA($H_{2}$ O) gal.	0.74	0.14	0.54	0.08	0.07
200 mA ${\text{cm}}^{-2}$	EIS	0.56	0.34	0.06	0.01	0.01
	CFRA($O_{2}$) gal.	0.99	0.62	0.02	0.02	0.01
	CFRA($H_{2}$ O) gal.	0.75	0.48	0.66	0.16	0.04
300 mA ${\text{cm}}^{-2}$	EIS	0.71	1.73	0.12	0.02	0.02
	CFRA(O (HTMLtranslationfailed)) gal.	0.99	2.41	0.1	0.05	0.04
	CFRA($H_{2}$ O) gal.	0.85	2.15	0.74	0.13	0.07

The mean square sensitivity to the cathode charge transfer coefficient does not significantly change across the steady state current densities for all FRA techniques. On the other hand, considering the analogous change of the operating conditions, the mean square sensitivity to the oxygen diffusivity in GDL increases for all the cases. Regarding the parameters related to the water transport, their influence on the EIS and CFRA using oxygen pressure inputs also increases along with the current densities. However, their impact is significantly less pronunced than the one they have on the CFRA through water pressure perturbations.

Usually, a threshold value for the mean square sensitivity below which the related parameters cannot be considered identifiable is fixed. Since they are used for comparison purpose in this work, this limit term is not defined.

The collinearity index has been determined for seven parameters subsets and at different operating currents as previously done in the sensitivity analysis. These subsets have been sorted out in order to measure the correlation between parameters that affect phenomena, whose effects may overlap within the frequency response spectra. As displayed in Table 3, the subset 1 contains the cathode charge transfer coefficient and the porosity related to the diffusion of oxygen in the GDL. The collinearity between the intrinsic diffusivity of water in Nafion and the electro-osmotic drag constant is determined in subset 2. The subsets from 3 to 6 are defined including all possible combinations of three parameters associated with the various mass transfer phenomena. Finally, the subset 7 contains all other parameters with the exception of the charge transfer coefficient.

**Table 3:** Parameters subsets considered for the determination of collinearity

Subset number	Parameters
1	$\alpha_{c}$, $\varepsilon_{O_{2}}$
2	$D_{H_{2}O}^{0,M}$, $K_{H^{+}}$
3	$\varepsilon_{O_{2}}$, $\varepsilon_{H_{2}O}$, $D_{H_{2}O}^{0,M}$
4	$\varepsilon_{O_{2}}$, $\varepsilon_{H_{2}O}$, $K_{H^{+}}$
5	$\varepsilon_{O_{2}}$, $D_{H_{2}O}^{0,M}$, $K_{H^{+}}$
6	$\varepsilon_{H_{2}O}$, $D_{H_{2}O}^{0,M}$, $K_{H^{+}}$
7	$\varepsilon_{O_{2}}$, $\varepsilon_{H_{2}O}$, $D_{H_{2}O}^{0,M}$, $K_{H^{+}}$

**Figure 4:**
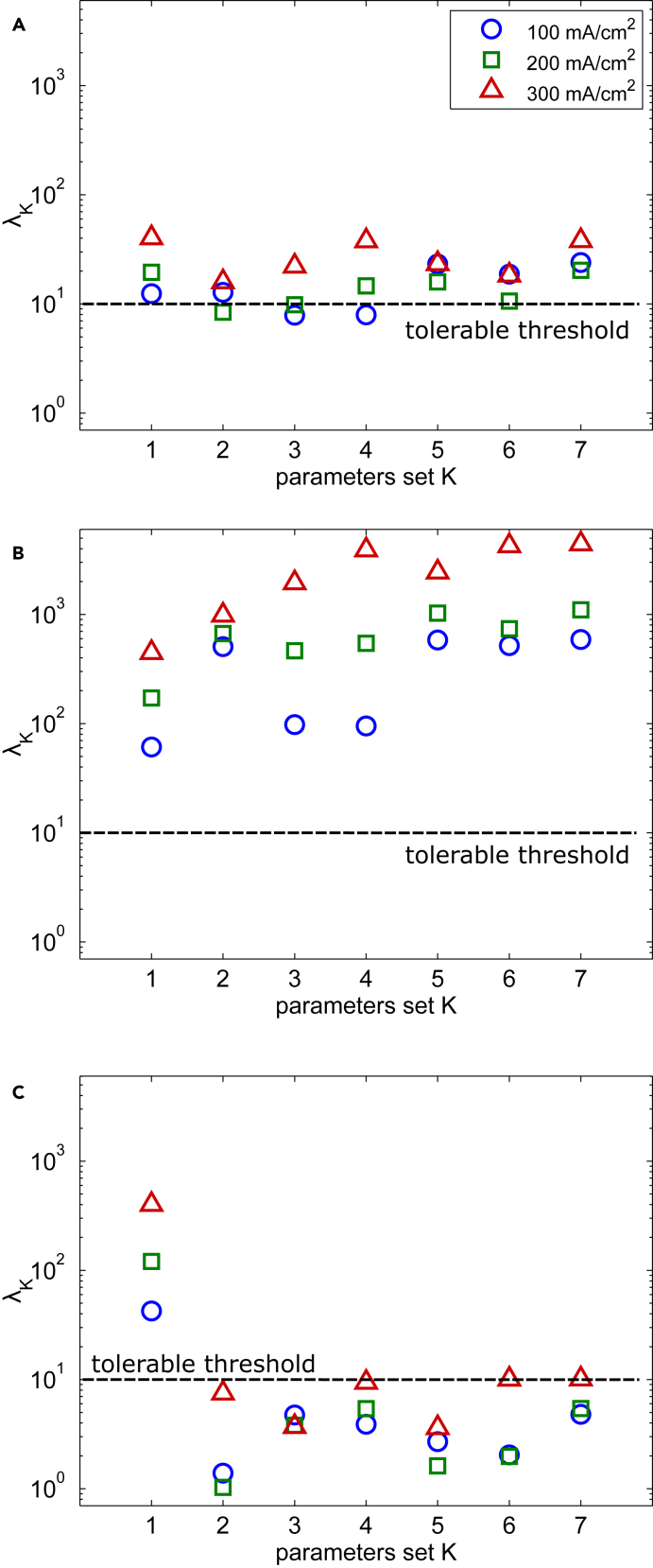
Collinearity index related to the different parameters subsets for the different FRA techniques and at different steady state currents (blue circles 100 mA ${\text{cm}}^{2}$, green squares 200 mA ${\text{cm}}^{2}$, and red triangles 300 mA ${\text{cm}}^{2}$) (A) EIS. (B) CFRA performed by using oxygen pressure input. (C) CFRA performed by using water pressure input.

The results of the collinearity analysis of the different subsets are reported in Figures 4A–4C where the tolerable limit of 10 is also indicated. The subset 1 shows remarkable correlation between its parameters, $\alpha_{c}$ and $\varepsilon_{O_{2}}$, regardless of the FRA technique and the operating current density. This implies the impossibility of identifying these parameters together through fitting. Therefore, one of them must be fixed in the model or determined through other experimental methods. This finding can be rationalized by reporting the following expression for the magnitude of $\zeta_{I,O_{2}}$ at low frequencies, which was derived in our previous works^17^^,^^39^:

(Equation 7) $$\zeta_{I,O_{2}}\left(\omega \right)\propto \frac{\text{RT}}{\alpha_{c}FP_{O_{2},\text{ss}}^{\text{CL}}}\text{.}$$

It is evident that this value is influenced by the charge transfer coefficient, denoted as $\alpha_{c}$, and the oxygen pressure within the catalyst layer, represented by $P_{O_{2},\text{ss}}^{\text{CL}}$. The latter parameter is directly connected to the porosity relative to the oxygen diffusion, $\varepsilon_{O_{2}}$. Consequently, for a given experimental value of $\zeta_{I,O_{2}}$ observed at low frequencies, an infinite number of combinations of $\alpha_{c}$ and $\varepsilon_{O_{2}}$ could potentially satisfy Equation 7. This implies that these parameters cannot be precisely determined.

Regarding the subsets 2–6, the EIS shows correlation indexes at the edge of the tolerable limit, but only at the current density values of 100 mA ${\text{cm}}^{-2}$ and 200 mA ${\text{cm}}^{-2}$ (Figure 4A). However, all the four transport parameters together (subset 7) are significantly interdependent at all operating conditions considered and, consequently, their estimated values would be unreliable.

The CFRA conducted with oxygen perturbations reveals collinearity indices significantly exceeding the established threshold across all subsets (see Figure 4B). This observation, supported by the sensitivity analysis results, suggests that while the FRA method primarily captures the dynamics of oxygen transport, parameters associated with other phenomena still quantitatively affect the transfer function’s values across the considered frequency range. The resulting high collinearity stems from the overlap of these influences, a topic we have elaborated on in our previous publication.^17^

Conversely, employing water pressure perturbations in CFRA experiments results in minimal parameter interdependence (Figure 4C). Notably, the collinearity indices fall well below the tolerance threshold for subsets 2 to 7. Furthermore, the impact of water diffusion and the electro-osmotic drag constant on the spectra are nearly orthogonal, especially as the optimal value for one parameter is determined within its subset (see subset 2).

In summary, the findings from the sensitivity and collinearity analyses can be encapsulated in the following key points.

(1) Despite all the FRA techniques present high sensitivity to $\alpha_{c}$ as well as to $\varepsilon_{O_{2}}$, their values cannot be correctly estimated together through fitting of a spectrum as these parameters are extremely correlated.
(2) Fitting of spectra of CFRA performed through oxygen pressure inputs does not provide a trustworthy parameter estimation due to the high collinearity index for all the parameters subsets investigated.
(3) Fitting of EIS spectra mostly imply an acceptable value of the collinearity indexes, but the sensitivity about parameters related to water transport in Nafion is low.
(4) CFRA performed through water pressure inputs is the most sensitive technique to parameters related to water transport in Nafion, and, consequently, it can be regarded as most suitable technique to detect membrane drying out.
(5) CFRA performed through water pressure inputs present the lowest collinearity indexes among the other FRA, and the optimal condition of orthogonality is approached in many subsets.

To conclude, according to the results obtained, the application of the CFRA through water pressure perturbation implies the highest parameters identifiability. Therefore, this technique can be regarded as the most trustworthy diagnostic tool in the operating condition explored.

### Validation of the identifiability analysis

In this section, the statements concluded through the identifiability analysis are validated. For this purpose, the considered model parameters are estimated by fitting the experimental Bode magnitude spectra. Their identifiability is proven by checking the convergence of the fitting algorithm to a stable solution. Moreover, it is verified that the estimated values of the parameters and their trends along the different operating conditions are in agreement with the physical understanding of a PEMFC.

The parameters are determined by finding the minimum of the residual sum of squares (RSS) expressed by the following equation:

(Equation 8) $$\text{RSS}={\left(H_{\exp}-H_{\text{sim}}\left(\theta \right)\right)}^{T}\left(H_{\exp}-H_{\text{sim}}\left(\theta \right)\right)\text{.}$$

The MATLAB function lsqnonlin is employed to perform the calculations. Upper and lower limits for each parameter are determined according to their physical meaning and are reported in the relative table in the STAR Methods section.

As concluded in the previous section, the charge transfer coefficient $\alpha_{c}$ cannot be identified together with parameters related to the mass transport. For this reason, its value was fixed. The ohmic free steady state voltage of the performed experiments lies between 659 mV and 762 mV. Under these conditions, a Tafel slope of 120 mV $s^{-1}$ is measured. This corresponds to a value of 0.5 for the charge transfer coefficient.^40^ Therefore, this quantity was set in the model simulations.

The fitting procedure was performed according to two steps in order to verify the collinearity among the parameters. At first, $\varepsilon_{O_{2}}$ and $\varepsilon_{H_{2}O}$ were estimated, while the parameters associated to the transport in Nafion $D_{\lambda }$ and $K_{H^{+}}$ were kept constant. Subsequently, the latter were fitted by holding the former at the new estimated values. Then, the procedure was repeated with the new dataset. In case of large interdependence between the two groups of parameters, different results were obtained after each run. A maximum of four repetitions were performed if no stable solutions were achieved. In that case, the parameters were considered not identifiable due to the large interdependence among each other.

The results collected according to the described procedure confirm the statements of the identifiability analysis. For instance, a unique set of parameters matching the spectra of the CFRA performed through oxygen pressure inputs could not be obtained due to the extensive collinearity among parameters. Regarding the EIS, a stable parameter set was achieved after two or three repetitions of the fitting procedure. Differently, as expected, a steady solution was easily achieved after the first run in the case of fitting of spectra relative to the CFRA through water pressure perturbation.

**Figure 5:**
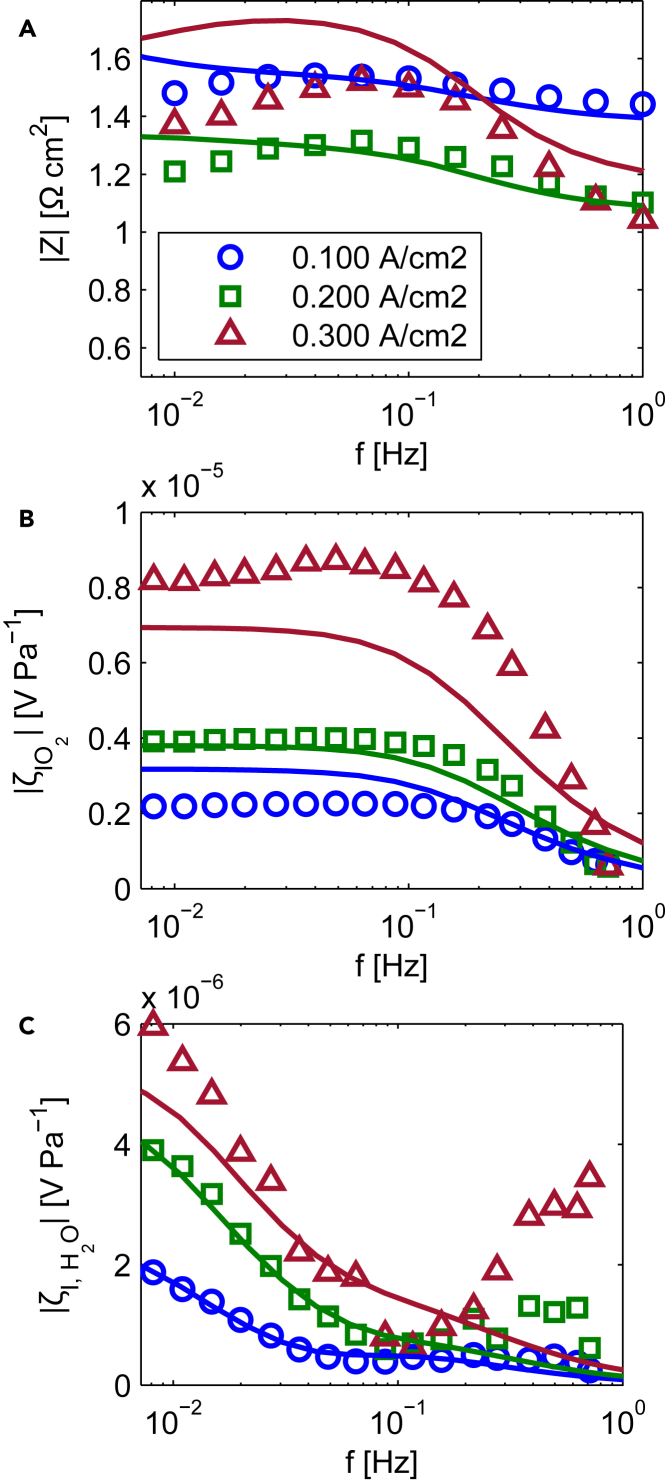
Comparison between fitted (solid lines) and experimental (circles, squared, and triangles markers) FRA spectra at different steady state current density (blue diagrams 100 mA ${\text{cm}}^{2}$, green diagrams 200 mA ${\text{cm}}^{2}$, and red diagrams 300 mA ${\text{cm}}^{2}$) (A) EIS. (B) CFRA performed by using oxygen pressure input. (C) CFRA performed by using water pressure input.

The fitted magnitude plots relative to the different FRA functions are reported in Figures 5A–5C together with the experimental ones. The parameters set obtained in the last fitting run is considered for the cases in which the algorithm was not converging. It is remarkable that the best match is reached for the spectra of the CFRA performed through water pressure inputs. Discrepancies between the model and the experiments are only observed at high frequencies for the spectra collected at 200 mA ${\text{cm}}^{-2}$. As discussed in our previous works, these could be due to phenomena that are not accounted in the model, for example, the effect of the distribution of the water perturbation along the cathode flow field or the transport of liquid water in the GDL.^17^^,^^39^ Therefore, an extension of the model including changes also in the channel coordinates (2-dimensional model), and mass balances for the liquid water in the GDL and catalyst layer that account for the influencing driving forces could reproduce such patterns.

Larger residues are observed for the other FRA methods. These discrepancies could be due to improper values of fixed parameters that present a high sensitivity and collinearity to the fitting parameters, such as $\alpha_{c}$. This is often verified in data fitting of model with a large number of fixed parameters. A more detailed discussion on this issue can be found in a study by Brun at al.^31^

**Figure 6:**
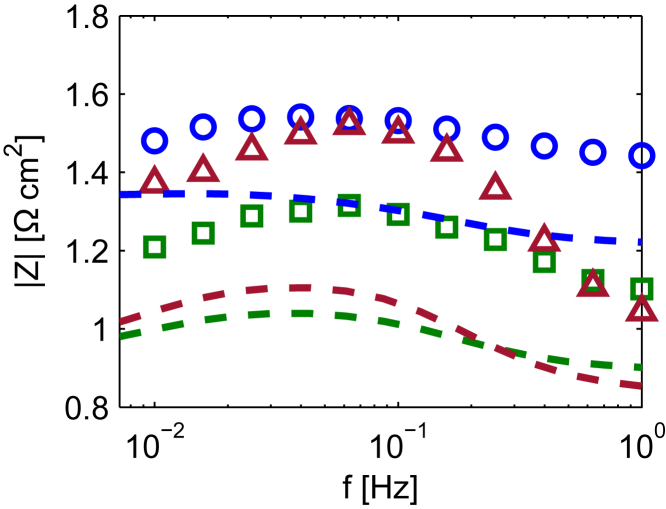
Comparison between experimental EIS spectra (circles, squares and triangles markers) and simulated ones (dashed lines) plotted by using the parameters set estimated through the fitting of the CFRA through water pressure inputs Blue diagrams 100 mA cm (HTML translation failed), green diagrams 200 mA ${\text{cm}}^{2}$, and red diagrams 300 mA ${\text{cm}}^{2}$).

The fitted EIS spectra deviate from the qualitative trend of the experiments at lower frequencies (Figure 5). This divergence could be attributed to the scarce identifiability of the parameters related to the transport in Nafion or, as previously mentioned, to improper values of fixed parameters. As noticeable by Figure 6, the same plots simulated by using the parameters set estimated by the fitting of spectra relative to CFRA through water pressure input reproduce the qualitative behavior correctly. On the other hand, the values of the experiments are underestimated. As the detection of correct qualitative trends along different operating conditions is sufficient for the establishment of a tool in a fault identification scheme, this result indicates the suitability of the water pressure perturbation for such application.

In Table 4, the parameters estimated through fitting of the FRA spectra at the different current densities are reported. In the case of non-convergence to a stable solution, the parameter set obtained by the fourth run of the estimation algorithm has been reported. Physically reasonable trends are obtained only in the case of CFRA through water pressure input. For instance, as expected, the porosity related to the oxygen diffusion $\left(\varepsilon_{O_{2}}\right)$ in GDL decreases along with the operating current density, since a higher amount of liquid water produced by the oxygen oxidation reaction (ORR) fills the pores and increases the mass transport resistance. The concurrent increase of the porosity related to the water diffusivity $\left(\varepsilon_{H_{2}O}\right)$ could also be physically meaningful, as the water is dragged out from the GDL not only through diffusion, but via permeation through the hydrophilic pores as well. Therefore, the overall transport flux of water rises.

**Table 4:** Parameters fitting the magnitude Bode plots related to the different FRAs techniques at different current densities

Current	Technique	$\varepsilon_{O_{2}}$	$\varepsilon_{H_{2}O}$	$D_{H_{2}O}^{0,M}/D_{H_{2}O}^{0,M*}$	$K_{H^{+}}/K_{H^{+}}^{*}$
100 mA ${\text{cm}}^{-2}$	EIS	0,03	1	0,1	1,85
	CFRA($O_{2}$) gal	0.69	0.03	0.1	0.91
	CFRA($H_{2}$ O) gal	0.15	0.78	0.6	2.45
200 mA ${\text{cm}}^{-2}$	EIS	0.34	0.06	0.01	0.01
	CFRA($O_{2}$) gal.	0.8	0.3	0.11	2.98
	CFRA($H_{2}$ O) gal.	0.06	1	1.32	2.3
300 mA ${\text{cm}}^{-2}$	EIS	0.032	0.48	1	1
	CFRA($O_{2}$)gal.	0.065	0.48	1.7	0.66
	CFRA($H_{2}$ O) gal.	0.045	0.97	2.97	2.42

The parameters $D_{H_{2}O}^{0,M}$ and $K_{H^{+}}$ are fitted with respect to their nominal value $D_{H_{2}O}^{0,M*}$ and $K_{H^{+}}^{*}$.

According to the model equations, the intrinsic diffusivity $D_{H_{2}O}^{0,M}$ is related to the Fickian diffusivity in Nafion $D_{H_{2}O}^{M}$ according to the proportional linear relationship proposed in the work of Fuller and displayed in Table 1. However, this expression is an approximation, as the experimental studies observe a nonlinear and non-monotonic relationship.^37^^,^^41^ Then, any change of the fitted value of $D_{H_{2}O}^{0,M}$ quantifies the deviation from this mathematical expression.

An increment of this parameter is obtained along with the current density. This behavior could be reasonable, as more water is produced and the membrane could be more hydrated meaning a higher water diffusivity. Nevertheless, as mentioned previously, the relationship between the diffusivity and water content in Nafion is more complex and not always proportional. Additionally, a higher current density does not necessarily imply a better membrane hydration, as the latter depends on the interplay of different mass flows counteracting each other, i.e., electro-osmotic flux, water back-diffusion, and the different transport mechanisms in the GDL. Therefore, proving the physical plausibility of the fitted parameters requires a thorough analysis, which is addressed further.

Most of the experimental studies in the literature report a linear relationship between the electro-osmotic drag flux and the water content, as the one used in the PEMFC model (see section mass balance in Nafion membrane in Table 1).^37^^,^^42^^,^^43^ Therefore, the fitted value for $K_{H^{+}}$ should be unchanged across the different operating condition. As noticeable, this is verified in the case of the CFRA performed by water pressure input (see Table 4).

**Figure 7:**
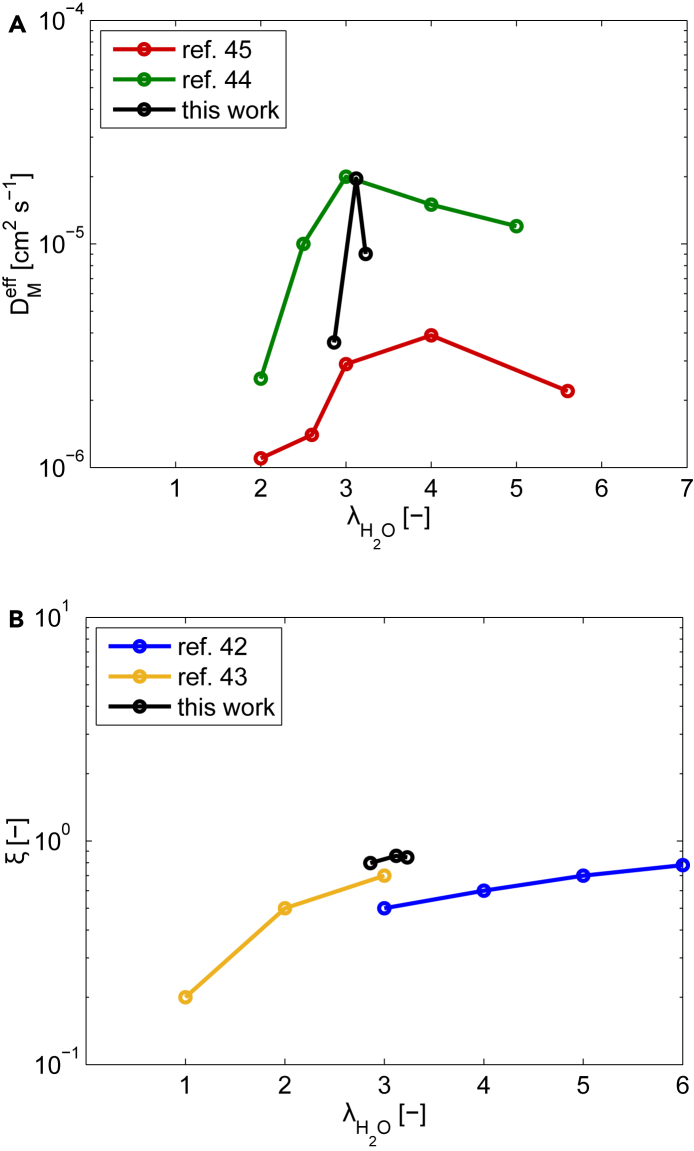
Estimated parameters related to the water transport in Nafion together with the ones measured in other studies (A) Fickean water diffusivity. (B) Electro-osmotic coefficient.

To further investigate the physical consistence of the calculated Fickian diffusivity and electro-osmotic drag coefficient in the Nafion membrane, these parameters are reported along with the average water content across the membrane length predicted by the model at the different current densities in Figure 7. Moreover, experimentally measured values reported in the literature are also displayed for comparison purposes. It must be pointed out that the measurements of such parameters reported in different studies can differ up to one order of magnitude. This is probably due to their sensitivity to operating conditions and experimental methods employed.^37^ However, there is an agreement on the qualitative trends observed. It is noticeable that the determined Fickian diffusivity presents a maximum at the intermediate current density of 200 mA ${\text{cm}}^{-2}$, corresponding to a water content $\lambda_{H_{2}O}$ of 3. This is also observed in the data of the study reported.^44^^,^^45^ Moreover, the fitted values lies in the same order of magnitude. The electro-osmotic coefficient also reproduces the qualitative trend of the experiments, with a quantitative discrepancy within one order of magnitude.

The parameters obtained by the fitting of the spectra of the EIS and CFRA performed using oxygen pressure perturbations do not present the expected trends. For example, an increase in the porosity related to oxygen diffusion in GDL, indicating an unrealistic improvement in mass transport conditions, is registered at higher current densities. Another contradictory trend is the change of the electro-osmotic constant $K_{H^{+}}$ up to one order of magnitude.

To conclude, the presented results show that the fitting of the spectra related to the CFRA performed by water pressure inputs provides physically consistent results. Therefore, this technique can be regarded as the most trustworthy FRA to be employed as a tool for fault identification among the others considered.

### Conclusions and future prospects

In this work, the employment of different FRA techniques as a tool for online monitoring in fault identification systems for PEMFCs was benchmarked. For this purpose, the trustworthiness of the parameters obtained by fitting of a validated PEMFC model to their spectra was evaluated by performing an identifiability analysis.

Classic EIS and the novel CFRAs based on oxygen and water pressure perturbations were considered as FRA techniques. The identifiability analysis clearly shows that the CFRA performed by water pressure input gives the most robust parameter estimation among the other FRA methods, and provides a unique set of parameter fitting the data. Moreover, the parameters obtained at different current densities give physically meaningful trends, correctly tracking the changes in mass transport resistance for oxygen and water in the GDL, as well as the water transport in the Nafion membrane. Additionally, the obtained qualitative behavior matches the one observed in experiments reported in the literature. Therefore, it is suggested that the use of water pressure in FRA for PEMFC diagnosis can be regarded as a promising strategy.

Finally, it is demonstrated that more care should be taken in verifying the quality of the parameter estimated, even though established experimental methods like EIS are employed. The presented analysis should be adopted routinely to test the reliability of diagnostic tools, or more generally, experimental routines.

### Limitation of the study

The study presented is limited to a specific range of operating conditions within which the employed model can accurately predict the dynamic behavior of PEMFCs. It excludes several potentially influential phenomena, particularly under other operating conditions. Notably, it does not consider phenomena such as platinum oxidation, peroxide formation, and effects pertaining to water transport in the GDL and the catalyst layer. Consequently, it is advisable to broaden the scope of the research to include additional operating inputs, such as varying feed humidities.

## STAR★Methods

### Key resources table

**Table:** 

REAGENT or RESOURCE	SOURCE	IDENTIFIER
**Software and algorithms**		

MATLAB	Mathworks	https://www.mathworks.com
NOVA	Metrohm	https://www.metrohm.com

**Others**		

MEA	Quintech	https://www.quintech.de
Gas diffusion layer	Toray	https://toray-cfe.com/
AUTOLAB	PGSTAT302	https://www.metrohm.com
Vaisala DMT340 dew point meter	Vaisala	https://www.vaisala.com/de
OXR430-UHS oxygen fiber sensor	Pyro Gmbh	https://www.pyroscience.com

**Data repository**		

Data related to experiments and simulations	Edmond	https://doi.org/10.17617/3.FGIUET

### Resource availability

#### Lead contact

Further information and request for resources should be directed and will be fulfilled by the lead contact, Antonio Sorrentino (sorrentino@mpi-magdeburg.mpg.de).

#### Materials availability

This study did not generate a new unique reagent.

#### Data and code availability

• Data: All the data discussed in the paper are available on the repository platform Edmond. The link for the dataset is listed in the key resources table.
• Code: The codes related to the model simulations are available upon reasonable request to the lead contact.
• Other: Any additional information required to reanalyze the data or code reported in this paper is available from the lead contact upon request.

### Method details

#### Fuel cell hardware

This study utilized a single PEMFC with a 25.8 ${\text{cm}}^{2}$ active area and parallel channel flow fields for all experiments. The membrane electrode assemblies (MEAs) were supplied by Quintech, and are constituted by an advanced surface area carbon coated on both sides of a Nafion 115 membrane. The platinum loading was 1 mg/${\text{cm}}^{2}$ on both the cathode and anode sides. The gas diffusion layer (GDL) utilized was Toray paper TGP-H-060. Temperature control of the fuel cell was achieved through a heating box with a PID controller. Fuel cell voltage and current were regulated and recorded using an Autolab potentiostat PGSTAT302 with an FRA unit and a BOOSTER20A during the experiments.

#### Experimental setup

The experimental setup resembles the standard setup for evaluating PEMFCs performance. It involves supplying dry streams of pure oxygen and nitrogen with 99.999 purity in air proportion, using a Bronkhorst EL Flow mass flow controller for precise dosing. These gases are humidified in a bubbler with an external jacket containing water heated by a heating circulator, ensuring a humidified gas stream at a set dew point temperature. To prevent water condensation, tube temperatures are maintained at least 10°C above the dew point using heating tapes with Pt100 sensors and PID controllers. Humidity and temperature are monitored by a Vaisala DMT340 dew point meter, and a sensor from Elettrotechnik Gmbh checks the inlet’s total pressure.

Oxygen and water partial pressures of the main feed are periodically altered by adding small dry oxygen or nitrogen flows via a switching valve, which alternates the flow direction to either the cell or the outlet. This setup allows for the regulation of pressure perturbation frequencies by adjusting the valve’s switching times. The added flow rate varies between 3 and 6 of the main feed to maintain the electric response’s linearity. Oxygen variations are detected by an OXR430-UHS oxygen fiber sensor (9) from Pyro Science Gmbh, placed before the cathode entrance.

For both CFRA and EIS measurements, it has been assured the stability of the system along the time of the experiments.

#### Simulations

The model developed is composed of six second-order partial differential equations (PDEs), which include five spatially distributed mass balances for the components of the gas mixture within the Gas Diffusion Layers (GDLs) three for the cathode side and two for the anode side. Additionally, there is a PDE concerning water transport in the membrane. To solve this set of equations, the formulation of twelve boundary conditions (BCs) is necessary. Moreover, the model incorporates two ordinary differential equations (ODEs) for the charge balance.

In order to solve numerically the model equations, each PDE has been discretized in a set of ODEs. After this step, a differential algebraic equations (DAEs) system was obtained (67 ODEs and 2 algebraic relations). It was solved in MATLAB environment using the ode15s solver. The number of discretization elements was determined by increasing the number of them until the solution was converging.

The procedure to obtain the sensitivity coefficient related to the different parameters analyzed is described in the section STAR Methods.

**Table:** List of parameters and their meaning

**Latin**		
$ai_{0}$	Product of specific catalyst area and current density	A $m^{-3}$
$A_{\text{cell}}$	Area of the electrode	$m^{2}$
$C_{\alpha }$	Concentration	mol $m^{-3}$
$C^{\text{DL}}$	Double layer capacity	F $m^{-2}$
$D_{\alpha }$	Diffusivity coefficient	$m^{2}$$s^{-1}$
E	Voltage	V
F	Faraday constant	C ${\text{mol}}^{-1}$
$F_{\alpha ,\text{in}}$	Inlet volumetric flow rate	$m^{3}$$s^{-1}$
$F_{\alpha ,\text{out}}$	Outlet volumetric flow rate	$m^{3}$$s^{-1}$
J	Current density	A $m^{-2}$
$J_{\alpha }$	Diffusive flux	mol $m^{-2}$$s^{-1}$
$K_{H^{+}}$	Electroosmotic drag constant	–
H	Generic transfer function	–
I	Imaginary unit	
I	Total current	A
L	Observation error vector	–
$m_{\text{EW}}$	Molecular weight of Nafion	Kg ${\text{mol}}^{-1}$
$N_{w}$	Water flux through the membrane	mol $m^{-2}$$s^{-1}$
$P_{\alpha }$	Partial pressure	Pa
$P_{\text{tot}}$	Total pressure	Pa
$r_{\text{ORR}}$	Reaction rate of oxygen reduction reaction	mol $m^{-2}$$s^{-1}$
R	Universal gas constant	J ${\text{mol}}^{-1}$$K^{-1}$
$S_{p}$	Sensitivity matrix	–
T	Time	s
$t^{\text{CL}}$	Catalyst layer thickness	m
T	Temperature	K
$\bar{u}$	Gas average velocity	m $s^{-1}$
V	Volume of the channel	$m^{3}$

**Greek**		

α	Charge transfer coefficient	–
ε	Porosity	–
$\zeta_{I}$	Galvanostatic CFRA transfer function	V ${\text{Pa}}^{-1}$
η	Overpotential	V
$\theta_{i}$	Generic parameter	–
$\kappa_{H^{+}}$	Proton conductivity in Nafion	S $m^{-1}$
$\lambda_{H_{2}O}^{M}$	Water content in Nafion	–
ξ	Electroosmotic drag force	–
$\rho_{\text{EW}}$	Density Nafion	kg $m^{-3}$
σ	Parameter variance	–
ω	Angular frequency	Hz
τ	Time constant	Hz
φ	Simulation vector	–
Φ	Ohmic voltage loss	V

**Superscripts**	

0	Nominal value
(HTMLtranslationfailed)	Anode
c	Cathode
CH	Channel
CL	Catalyst layer
DL	Double layer
eff	Effective value
GDL	Gas diffusion layer
M	Nafion membrane

**Subscripts**	

C	Critical value
diff	Parameter related to diffusion process
i	Index
In	Inlet
J	Index
K	Index
Kin	Kinetic parameter
K	Parameters subset
M	Index
N	Index
ORR	Oxygen oxidation reaction
out	Outlet
ref	Reference value
Set	Set operating value
W	Water
α	General component

**Table:** Values of the model parameters

Quantity	Value
$ai_{0}$ Product of specific catalyst area and exchange current (A $m^{-3}$)	$10^{4}$
$A_{\text{cell}}$ Geometric surface area of the catalyst ($m^{2}$)	$2.6\times 10^{-3}$
$C^{\text{DL}}$ Double layer capacitance for cathode and anode (F $m^{-2}$)	500
$C_{O_{2},\text{ref}}$ Reference concentration $O_{2}$ 2 (mol $m^{-3}$)	40
$D_{H_{2}O,\text{air}}$ Diffusivity for $H_{2}$ O ( $m^{2}$$s^{-1}$)	$1.14\times 10^{-5}$
$D_{N_{2},\text{air}}$ Diffusivity of $N_{2}$ ($m^{2}s^{-1}$)	$3.07\times 10^{-5}$
(HTMLtranslationfailed) Diffusivity of $O_{2}$ ($m^{2}$$s^{-1}$)	$1.42\times 10^{-5}$
$D_{H_{2}O}^{0,M}$ Nominal diffusivity of water in Nafion ($m^{2}$$s^{-1}$)	$2.1\times 10^{-7}$
$E_{\text{ocp}}$ Open circuit potential (V)	1.2
$F_{\text{in},c}$ Gas flow rate at cathode ($m^{3}$$s^{-1}$)	$6.94\times 10^{-6}$
$F_{\text{in},a}$ Gas flow rate at anode ($m^{3}$$s^{-1}$)	$4.4\times 10^{-6}$
$K_{\text{ad},H_{2}}$ Kinetic constant for hydrogen adsorption (Pa)	$5.07\times 10^{4}$
$K_{\text{HOR}}$ Kinetic constant for hydrogen oxidation (mol $m^{-2}$$s^{-1}$)	$4.15\times 10^{-1}$
$K_{H^{+}}$ Electrosmotic drag constant	2.5
$P_{\text{tot}}$ Total pressure (Pa)	$10^{5}$
$P_{O_{2},\text{in}}^{\text{CH}}$ Oxygen pressure of the cathode inlet (Pa)	$1.58\times 10^{4}$
$P_{H_{2},\text{in}}^{\text{CH}}$ Hydrogen pressure of the anode inlet (Pa)	$7.51\times 10^{4}$
$t_{c}^{CL}$ Cathode catalyst layer thickness (m)	$10^{-6}$
T Temperature (K)	353
$T_{dp,a}$ Dew point temperature anode (K)	328
$T_{\text{dp},c}$ Dew point temperature cathode (K)	328
V Volume of the flow field ($m^{3}$)	$7.2\times 10^{-6}$
$\alpha_{a}$ Charge transfer coefficient at the anode	0.5
$\alpha_{c}$ Charge transfer coefficient at the cathode	0.55
$\varepsilon_{H_{2}O}$ Porosity of the GDL related to $H_{2}$ O	0.3
$\varepsilon_{O_{2}}$ Porosity of the GDL related to $O_{2}$	0.034
$\Lambda_{C}$ Stoichiometry factor at cathode $\left(300\text{mAc}m^{-2}\right)$	12

**Table:** Upper and lower boundaries set in the parameter estimation algorithm

Parameter	Lower value	Upper value
$\varepsilon_{O_{2}}$	0.03	1
$\varepsilon_{H_{2}O}$	0.03	1
$D_{H_{2}O}^{0,M}/D_{H_{2}O}^{0,M*}$	0.01	60
$K_{H^{+}}/K_{H^{+}}^{*}$	0.01	60

### Quantification and statistical analysis

The parameter estimation was performed in MATLAB environment using the solver lsqnonlin. No smoothing algorithm was applied to the data.
